# Aortic Arch and Frozen Elephant Trunk Surgery: Anesthetic Challenges and Strategies for Organ Protection

**DOI:** 10.3390/jcm15020877

**Published:** 2026-01-21

**Authors:** Debora Emanuela Torre, Carmelo Pirri

**Affiliations:** 1 Intensive Care Unit and Cardiac Surgery, Department of Cardiac Anesthesia, Ospedale dell’Angelo, 30174 Venice, Italy; deboraemanuela.torre@aulss3.veneto.it; 2 Department of Neurosciences, Institute of Human Anatomy, University of Padova, 35121 Padua, Italy

**Keywords:** frozen elephant trunk (FET), Aortic arch surgery, FET anesthesia, neuroprotection, organ protection, hypothermic circulatory arrest

## Abstract

**Background**: Aortic arch surgery using the frozen elephant trunk (FET) technique remains one of the most complex scenarios in cardiac anesthesia. The anesthesiologist plays a central role in maintaining neuroprotection, organ perfusion and hemodynamic stability during hypothermic circulatory arrest and selective cerebral perfusion. This review summarizes key anesthetic principles aimed at improving neurologic and systemic outcomes. **Methods**: This narrative review examines current evidence and expert recommendation on temperature and perfusion management, neuromonitoring, coagulation control and postoperative strategies specific to FET procedures. **Results**: Modern approaches emphasize moderate hypothermia with tailored selective cerebral perfusion, multimodal neuromonitoring and structured organ protection bundles. Evidence supports the use of physiology-guided perfusion, viscoelastic-based coagulation management and coordinated teamwork with surgical and perfusion specialists to reduce neurologic injury, bleeding and postoperative organ dysfunction. **Conclusions**: Anesthetic management in FET surgery requires an integrated, physiology-based strategy supported by advanced monitoring and close interdisciplinary coordination. Adoption of standardized organ-protection and perfusion protocols is essential to optimize neurologic and systemic outcomes in this high-risk population.

## 1. Introduction

Aortic arch pathology remains among the most demanding conditions in cardiovascular surgery and perioperative care. Acute type A aortic dissection, complex arch aneurysm, chronic post-dissection states and penetrating ulcers carry substantial mortality and neurological risk, particularly when the descending thoracic aorta is involved. The frozen elephant trunk (FET) procedure has become a key hybrid strategy, enabling single-stage total arch replacement with simultaneous endovascular treatment of the proximal descending aorta. While this approach improves distal remodeling and facilitates subsequent interventions, it also exposes patients to profound physiological stress that requires highly specialized anesthetic management [1,2,3]. The procedure is now predominantly carried out using dedicated hybrid systems, most commonly the Thoraflex Hybrid and the E-vita Open Neo, each integrating a surgical arch graft with a distal stented segment to stabilize the descending aorta. Their design has streamlined total arch replacement, improved distal sealing and enhanced consistency of perfusion management during circulatory arrest [4,5,6].

FET surgery differs fundamentally from proximal aortic repair because it entails deep or moderate hypothermic circulatory arrest, selective antegrade cerebral perfusion (SACP), complex cannulation strategies and deployment of a stented graft in the descending aorta [3,7,8]. Each phase alters cerebral, spinal, renal and visceral perfusion, demanding continuous integration of neuromonitoring, hemodynamic surveillance, metabolic assessment and transesophageal echocardiography. This physiology-guided approach allows real-time adjustment of perfusion flows, pressures, temperature and CO_2_ management to reduce ischemic and embolic injury. The shift from deep to moderate hypothermia has further reshaped intraoperative strategies. When paired with optimized SACP, moderate hypothermia reduces coagulopathy, inflammation and cardiopulmonary-bypass duration, although it requires meticulous control of metabolic suppression and rewarming to prevent neurological complications [9,10].

Beyond cerebral protection, FET procedures pose notable risks to the spinal cord, kidneys and splanchnic organs due to segmental artery coverage, ischemia–reperfusion injury and low-flow states. Effective management includes maintaining adequate hemoglobin and mean arterial pressure, tailoring perfusion techniques and employing early detection tools [11,12,13,14]. Coagulation disturbances remain a major contributor to morbidity, owing to hypothermia, hemodilution and extensive surgical surfaces. Viscoelastic-guided transfusion, fibrinogen optimization, antifibrinolytic therapy and blood-sparing measures are now essential components of contemporary care [15,16].

Postoperatively, patients require strict monitoring for neurological deficits, spinal cord ischemia, vasoplegia, acute kidney injury and coagulopathy. Avoiding secondary insults, hypotension, hypothermia and hyperoxia is critical for minimizing reperfusion injury and supporting recovery.

Overall, successful FET anesthesia relies on coordinated physiology-based, multidisciplinary management. This review synthesizes current evidence in temperature and perfusion strategies, neuromonitoring, organ protection, coagulation control and postoperative care, offering a structured framework to guide anesthesiologist through the complex physiological demands of FET surgery.

## 2. Materials and Methods

This narrative review was conducted to synthesize current evidence on anesthetic management strategies for aortic arch surgery performed with the FET technique. The review followed established methodological principles for non-systematic, evidence-based narrative synthesis. A comprehensive literature search was performed in PubMed/MEDLINE, Scopus and Web of Science, covering publications from their inception to November 2025. The search strategy included combinations of the following terms: “Frozen Elephant Trunk”, “aortic arch surgery”, “selective cerebral perfusion”, “hypothermic circulatory arrest”, “organ protection” and “neuromonitoring”. Reference lists of key articles and recent guidelines were also screened to identify additional relevant studies. Eligible sources included peer-reviewed clinical studies, randomized trials, observational cohorts, expert consensus statements, perfusion and surgical guidelines and relevant mechanistic or physiological studies. Articles were included if they provided data or expert insight into anesthetic management, perfusion strategies, neuromonitoring, coagulation or postoperative care for FET or aortic arch procedures. Publications focusing exclusively on surgical outcomes without anesthetic relevance and non-peer-reviewed material were excluded. Given the heterogeneity of available evidence, spanning clinical series, physiological studies and expert recommendations, no formal quantitative synthesis or risk-bias assessment was performed. Instead, findings were integrated through qualitative thematic analysis, emphasizing consistent patterns across studies, areas of consensus and clinically relevant trends in modern perioperative practice. This methodology allowed the development of a structured, physiology-oriented synthesis aligned with contemporary anesthetic practice in FET surgery.

## 3. Key Domains of Perioperative Anesthetic Management in FET Surgery

### 3.1. Surgical and Physiologic Considerations in FET Procedures

The frozen elephant trunk (FET) procedure combines total aortic arch replacement with deployment of a stented graft into the proximal descending thoracic aorta. This hybrid approach profoundly alters circulatory physiology and requires the anesthesiologist to anticipate rapid shifts in perfusion territories, systemic vascular resistance and metabolic demand. Manipulation of the arch, cross-clamping and the introduction of a rigid hybrid prosthesis expose the patient to varying degrees of hypoperfusion, embolic risk and reperfusion injury. In addition, covering intercostal and segmental arteries with the endovascular component may compromise spinal cord blood flow, creating a hemodynamic milieu in which even subtle reductions in mean arterial pressure or hemoglobin can precipitate neurological injury. The anesthetic strategy must therefore integrate surgical timing, perfusion objectives and organ-specific vulnerabilities. Understanding how each phase of the operation alters cerebral autoregulation, spinal cord perfusion pressure, renal delivery of oxygen and splanchnic flow is essential for maintaining physiological stability throughout circulatory arrest and rewarming [17,18].

#### 3.1.1. Cannulation Strategies and Their Hemodynamic Implications

The choice of arterial inflow site represents one of the principal determinants of perfusion balance during FET surgery, influencing cerebral flow distribution, embolic risk and the interpretation of arterial pressure monitoring throughout cardiopulmonary bypass and selective antegrade cerebral perfusion [19] (Table 1).

From an anesthetic perspective, the primary relevance of cannulation strategy lies not only in establishing adequate cerebral and systemic perfusion but also in shaping the reliability of hemodynamic monitoring and the detection of perfusion mismatches during cooling, circulatory arrest and rewarming. During selective antegrade cerebral perfusion (SACP), right radial arterial pressure may overestimate true cerebral perfusion pressure in unilateral cannulation, particularly in the presence of incomplete Willisian collateralization. Conversely, femoral arterial pressure may underestimate proximal aortic pressure when retrograde or dual-inflow perfusion configurations are used, owing to flow gradients and altered vascular resistance across the reconstructed arch. These physiological considerations support the routine adoption of dual-site arterial pressure monitoring, typically combining upper-limb (radial) and lower limb (femoral) line, to promptly identify discrepancies between cerebral and systemic perfusion territories [32,33,34]. Beyond pressure interpretation, cannulation strategy also modulates embolic risk and distal organ perfusion during circulatory arrest. Antegrade inflow configurations minimize retrograde mobilization of aortic debris toward cerebral vessels, whereas retrograde perfusion may expose the brain to higher embolic load, particularly in the presence of atherosclerotic or dissected descending aorta [20,21,22,23,27,28]. In complex reconstructions or pre-existing malperfusion, combined inflow strategies may provide more balanced whole-body perfusion, albeit at the cost of increased technical complexity and monitoring demands [31].

#### 3.1.2. Cardiopulmonary Bypass and Perfusion Configurations

CBP configurations in FET surgery must balance cerebral protection, systemic perfusion adequacy and technical constraints imposed by aortic arch reconstruction. Standard CBP is typically initiated with antegrade arterial inflow (axillary or innominate), combined with bicaval or single caval venous drainage, depending on surgeon preference [19]. During selective antegrade cerebral perfusion (SACP), contemporary practice generally targets flow rates of 6–10 mL/kg/min with perfusion pressures maintained around 35–50 mmHg. These parameters are adjusted according to neuromonitoring trends, temperature management and individual cerebrovascular anatomy, avoiding excessive pressures that may predispose to hyperperfusion injury, edema or embolic phenomena. Bilateral perfusion may be required when unilateral delivery produces asymmetrical NIRS declines or when circle of Willis competence is uncertain [35,36,37,38]. Lower body perfusion is suspended during total arch reconstruction, creating a predictable ischemia–reperfusion cycle for the viscera, kidneys and spinal cord. This period emphasizes the need for controlled metabolic suppression, stable perfusion before arrest, strict temperature regulation and careful management of rewarming once distal perfusion is restored [39,40].

#### 3.1.3. Physiological Transitions: Cooling, Arrest, Reperfusion, Rewarming

FET surgery involves some of the most dramatic physiological transitions encountered in cardiac anesthesia.

Cooling reduces cerebral metabolic rate and allows safe circulatory arrest but also induces coagulopathy, increases blood viscosity and alters drug pharmacokinetics; although hypothermia per se may promote hemoconcentration through increased viscosity and peripheral vasoconstriction, during cardiopulmonary bypass, these effects are typically outweighed by CBP-related hemodilution, resulting in a net dilutional state. The anesthesiologist must maintain controlled gradients between core and peripheral temperatures to avoid cerebral “temperature overshot” and ensure adequate oxygen delivery prior to arrest [41].

Circulatory arrest abolishes systemic perfusion to all organs except the brain when SACP is used. The priority becomes minimizing arrest duration, maintaining targeted cerebral perfusion and ensuring optimal metabolic suppression through temperature, anesthetic depth and CO_2_ strategy [36].

Reperfusion introduces flow to previously ischemic tissues and is associated with abrupt metabolic shifts, lactate release, endothelial activation and inflammatory response. Sudden increases in venous return can also challenge right ventricular function, particularly in patients with preexisting dysfunction [42,43].

**Figure 1:**
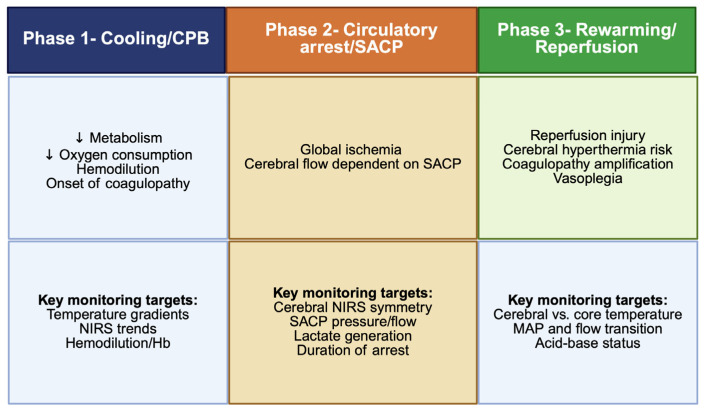
Temporal phases of FET surgery and related physiological implications. CPB: cardiopulmonary bypass; SACP: selective anterograde cerebral perfusion; MAP: mean arterial pressure; NIRS: near-infrared spectroscopy. Created in BioRender. Pirri, C. (2026) https://BioRender.com/daedw37 (accessed on 17 December 2025).

Rewarming must be progressive and tightly controlled. Rapid gradients may precipitate gas emboli, cerebral hyperthermia or excessive vasodilation leading to hypotension and impaired spinal cord perfusion. Acid-base correction, electrolyte management, hemoglobin optimization and precise vasopressor titration are critical during this phase. Overall, the success of FET surgery relies on anticipating these physiologic inflection points and maintain fine-tuned, real-time control over temperature, perfusion pressures, oxygen delivery and metabolic support [44,45,46], (Figure 1).

### 3.2. Temperature Management in Aortic Arch and FET Surgery

Temperature modulation remains a central component of organ protection during aortic arch and FET procedures. Hypothermia provides metabolic suppression, mitigates the biochemical cascade associated with ischemia and extends the safe duration of circulatory arrest. Yet, the benefits of reduced metabolic demand must be weighed against the physiological burdens imposed by cooling, coagulopathy, increased blood viscosity, altered enzymatic kinetics and destabilization of autoregulatory mechanisms. Effective temperature management therefore requires a meticulous balance between neuroprotection and the systemic consequences of hypothermia, guided by continuous integration of perfusion targets, neuromonitoring and dynamic hemodynamic assessment [47,48,49].

#### 3.2.1. Rationale for Hypothermia

Hypothermia offers neuroprotection primarily through the reduction in cerebral metabolic rate for oxygen (CMRO_2_), which declines exponentially with decreasing temperature [47]. This reduction stabilizes neuronal membrane potentials, slows adenosine triphosphate depletion, attenuates excitotoxic neurotransmitter release and limits free radical-mediated injury. Lower temperatures also suppress inflammatory and apoptotic pathways that otherwise intensify during ischemia–reperfusion [50]. Consequently, the depth of cooling must reflect a deliberate assessment of anticipated circulatory-arrest duration, reliability of cerebral perfusion pathways and patient-specific cerebrovascular physiology [51].

#### 3.2.2. From Deep to Mild Hypothermia: Evidence and Outcomes

Deep hypothermic circulatory arrest (DHCA) at 15–18 °C historically represented the standard for aortic arch procedures. Although it provides profound metabolic suppression, it is associated with prolonged cardiopulmonary bypass duration and greater systemic physiological burden [52,53,54]. The adoption of selective antegrade cerebral perfusion (SACP) has enabled safe transition toward moderate hypothermia (22–28 °C), which achieves comparable neurologic protection while improving systemic stability [55,56]. More recently, increasing experience with reliable unilateral or bilateral SACP has allowed selected centers to employ mild hypothermia (28–32 °C) for shorter arrest intervals, maintaining adequate cerebral metabolic suppression while reducing global physiological stress [57,58,59].

For most contemporary FET procedures, moderate hypothermia combined with well-regulated SACP and multimodal neuromonitoring provides optimal balance between cerebral protection and systemic homeostasis.

Emerging perfusion strategies have further expanded this paradigm. A 2025 series by Berretta et al. [60] reported the feasibility of performing selected FET procedures under full normothermia without circulatory arrest, using continuous antegrade perfusion to maintain cerebral and visceral flow throughout the reconstruction. Although applicable only to carefully chosen anatomies, this approach underscores the progressive shift toward minimizing hypothermia-related physiological burdens and highlights a potential future direction for temperature management in arch surgery.

#### 3.2.3. Temperature Targets, Gradients and Safety Parameters

The effectiveness of hypothermia depends not only on the target temperature but also on the manner in which is achieved. Cooling should proceed gradually, maintaining a core-peripheral gradient typically ≤10 °C to avoid heterogeneous cerebral cooling, vasoconstriction or localized thermal shifts that may distort neuromonitoring signals [44]. Common intraoperative targets include

- 24–28 °C for most FET procedures performed with bilateral or robust unilateral SACP [61];
- 20–22 °C in selected cases requiring extended arrest or when cerebral perfusion pathways are uncertain [62];
- In high-volume centers, mild hypothermia (28–30 °C) is selectively employed for shorter circulatory arrest intervals when SACP flow, neuromonitoring integrity and perfusion pressures are consistently reliable, thereby reducing overall physiological burden and CPB duration while maintaining uniform cerebral and systemic thermal profile [63,64].

Avoiding excessive cerebral cooling is critical, as disproportionate decreases in nasopharyngeal temperature relative to core temperature may impair autoregulation and predispose to hyperviscosity. Throughout cooling and low-flow states, attention to perfusate temperature, CO_2_ management and real-time neurologic monitoring is essential to ensure uniform thermal distribution and stable perfusion dynamics [44,51,65,66].

#### 3.2.4. Rewarming Strategies and Reperfusion Injury Prevention

Rewarming represents one of the most vulnerable phases of arch surgery, coinciding with loss of autoregulation, endothelial dysfunction and re-establishment of systemic flow after ischemic intervals. Uncontrolled rewarming can intensify oxidative stress, favor cerebral hyperthermia and precipitate hemodynamic instability [67,68]. Key principles include:

- Limit rewarming rates to ≤0.5 °C/min [44];
- Maintaining a core-perfusate gradient ≤ 4 °C [44];
- Avoiding arterial outlet temperatures > 37 °C to prevent cortical overheating [44];
- Titrating vasopressors judiciously to counteract hypothermia-related vasoconstriction followed by vasodilatory rebound [69];
- Preventing hyperoxia and correcting metabolic derangements before full reperfusion [70].

These measures help attenuate reperfusion injury and support stable cerebral and systemic hemodynamics during the transition to normothermia.

#### 3.2.5. Acid-Base Management (Alpha-Stat vs. pH-Stat)

Acid-base strategy during hypothermic cardiopulmonary bypass significantly influences cerebral physiology [71]. Alpha-stat management, which maintains blood gases uncorrected for temperature, preserves cerebrovascular autoregulation and cellular charge neutrality. It is generally preferred in adult aortic arch surgery, particularly when SACP is employed [71,72].

In contrast, pH-stat, by correcting gases to actual patient temperature, induces cerebral vasodilation and increases blood flow heterogeneity. While this may improve cooling uniformity in pediatric or deep hypothermic settings, in adult FET surgery, it may increase embolic risk and impair hemodynamic precision [72]. For these reasons, alpha-stat remains the favored approach in adults, allowing more reliable coupling between metabolic demand and cerebral blood flow during both cooling and rewarming [72]. Nevertheless, the lack of randomized controlled data in adult FET cohorts should prompt acknowledgement that this recommendation is based principally on physiological rationale, historic experience and expert consensus rather than on robust contemporary evidence. As such, acid-base strategy should always be integrated with other neuroprotective measures in a comprehensive perfusion protocol.

### 3.3. Cerebral Protection and Neuromonitoring

Cerebral protection is a central priority during aortic arch and FET surgery, given the vulnerability of the brain to ischemia, embolization and reperfusion injury [73]. The interplay between selective antegrade cerebral perfusion (SACP), hypothermia, perfusion pressures, embolic load and autoregulatory capacity requires an integrated, physiology-oriented strategy. Neuromonitoring serves as the real-time interface between perfusion mechanics and cerebral physiology, enabling dynamic titration of flow, pressure, temperature and acid-base management throughout the operative course [74].

#### 3.3.1. Selective Antegrade Cerebral Perfusion (SACP) Techniques

Selective antegrade cerebral perfusion (SACP) has become the principal strategy for cerebral protection during aortic arch and FET surgery, largely replacing deep hypothermic circulatory arrest and stand-alone technique. SACP preserves cerebral oxygen delivery by providing continuous, oxygenated blood flow to the carotid and vertebral territories during arch reconstruction. Its efficacy depends on the adequacy of inflow, distribution across the cerebral vasculature and compatibility with the patient’s cerebrovascular anatomy [75,76]. Two main configurations are employed: unilateral and bilateral SACP. Unilateral perfusion, typically via the right axillary or innominate artery, is technically simple and generally sufficient when the circle of Willis is complete. However, its effectiveness may be compromised in patients with anatomic variants, carotid stenosis or incomplete collateral pathways. Asymmetrical declines in near-infrared spectroscopy (NIRS) or unilateral EEG suppression often signal the need to convert to bilateral perfusion [77,78].

Bilateral SACP involves separate perfusion of both carotid arteries, either through direct cannulation or via branch grafts of the arch prosthesis. It provides more homogeneous cerebral perfusion and is particularly valuable in cases of uncertain cerebrovascular anatomy, pre-existing cerebrovascular disease or prolonged circulatory arrest. The trade-off is greater technical complexity and the need for careful flow balancing between hemispheres [78,79].

Perfusion flow during SACP is commonly initiated within ranges reported in the literature (approximately 6–10 mL/kg/min or equivalent absolute flows), but no universally accepted standards exist [35,80]. Flows are therefore titrated according to real-time neuromonitoring, primarily NIRS and EEG, to ensure adequate cerebral delivery while avoiding hyperperfusion [36]. Similarly, perfusion pressures are generally maintained within moderate ranges (often 25–50 mmHg) yet must remain adjustable based on individual cerebrovascular physiology and monitored cerebral responses [10]. CO_2_ management further influences cerebral blood flow as follows: mild hypercapnia may enhance perfusion homogeneity, whereas excessive hypocapnia can precipitate vasoconstriction and regional ischemia. Overall, flow, pressure and CO*_2_* targets should be individualized rather than fixed, guided continuously by neurologic and perfusion monitoring [81].

#### 3.3.2. Monitoring Cerebral Perfusion: NIRS, EEG, TCD

Effective neuromonitoring is essential during aortic arch and FET surgery, where cerebral perfusion depends on selective antegrade flow, hypothermia and highly dynamic changes in cerebrovascular resistance. No single modality captures all dimensions of cerebral physiology; thus, a multimodal strategy is required to detect hypoperfusion, maldistribution, embolic events and impaired autoregulation during cooling, arrest and rewarming [82].

- NIRS: near-infrared spectroscopy provides continuous, non-invasive assessment of regional cerebral oxygen saturation (rSO*_2_*) by measuring the relative balance of oxygenated and deoxygenated hemoglobin within the frontal cortex. Because rSO*_2_* reflects both cerebral blood flow and metabolic demand, NIRS is highly sensitive to perfusion changes during circulatory arrest and SACP. Typical intervention thresholds include a 20–25% decrease from baseline or absolute values falling below 50%. Decreases in rSO*_2_* may indicate insufficient SACP flow or pressure, impaired cerebral autoregulation, asymmetric perfusion during unilateral SACP or embolic obstruction. Conversely, abrupt increases, especially during rewarming, may signal loss of autoregulation or early hyper-perfusion. While NIRS is limited to superficial cortical territories and can be influenced by extracranial factors, its rapid responsiveness makes it indispensable for real-time titration of perfusion and ventilatory parameters [83,84].
- Electroencephalography (EEG) and processed EEG: offers complementary insight into cortical electrical activity, anesthetic depth and metabolic suppression. During controlled cooling, EEG progressively transitions through reduced amplitude, burst suppression and eventually electrocerebral silence, which confirms adequate metabolic depression before circulatory arrest. Asymmetric EEG suppression or re-emerge of electrical activity during arrest may indicate malperfusion or warming artifacts. Intraoperative seizures, although uncommon, may also be identified. Processed EEG modalities, such as bispectral index or spectral edge frequency, provide simplified, continuous indices that reflect global cortical activity. Although less detailed than a full montage, they can reliably indicate inadequate metabolic suppression, excessive anesthetic depth or ischemic changes, particularly when paired with NIRS [85,86,87].
- Trans-cranial Doppler (TCD): allows real-time evaluation of blood flow velocity in intracranial vessels, typically the middle cerebral artery. During SACP, TCD can verify antegrade flow delivery, detect perfusion asymmetries and identify transient drops in velocity consistent with ischemia or vessel obstruction. One of the unique strengths of TCD is its ability to detect high-intensity transient signals (HITSs), which indicate gaseous or particulate microemboli. This is particularly relevant during manipulation of the arch branches, deployment of the stented graft and initiation of bypass, when embolic load may be highest. Although TCD is operator dependent and its feasibility is limited by acoustic windows, its capability to detect embolic phenomena and flow directionality provides information not obtainable from NIRS or EEG [83,88,89].

#### 3.3.3. Prevention of Embolic and Hypoperfusion Injury

During arch manipulation, cannulation and deployment of the stented graft, the risk of particulate and gaseous embolization is substantial. Retrograde perfusion, particularly when femoral cannulation is used, may mobilize atheroma or debris from the descending aorta into the cerebral circulation. Additional embolic sources include incomplete deairing, residual intracardiac air, thrombus formation in low-flow regions and manipulation of calcified branch vessels [90,91,92,93,94]. Preventive strategies include antegrade perfusion whenever feasible, which reduces retrograde embolic load; meticulous deairing and venting techniques, particularly during rewarming and before clamp removal; minimizing aortic manipulation prior to cooling, to avoid mobilization of debris under normothermic conditions; CO*_2_* flooding of the operative field which reduces the volume of entrained air and its embolic potential; use of transesophageal echocardiography and, when feasible, TCD to detect air or particulate emboli and guide corrective interventions. During stent deployment in FET surgery, slow, controlled release of the graft and avoidance of sudden pressure surges help limit plaque disruption and embolic displacement [95,96].

Cerebral hypoperfusion can occur during cooling, circulatory arrest or selective antegrade cerebral perfusion. Factors contributing to inadequate flow include insufficient perfusion pressure or flow, excessive vasoconstriction during hypothermia, incomplete circle of Willis and unrecognized carotid or vertebral disease [74,79].

#### 3.3.4. Pharmacologic Neuroprotection

Pharmacologic neuroprotection serves as an adjunct to perfusion-based and temperature-mediated strategies, aiming to attenuate the biochemical and electrophysiologic consequences of cerebral ischemia and reperfusion during aortic arch and FET surgery. Although no pharmacologic agent alone is sufficient to prevent neurologic injury, several classes of drugs provide targeted modulation of excitotoxicity, oxidative stress, inflammation and metabolic demand, complementing the mechanical protection achieved with selective antegrade cerebral perfusion and hypothermia [97]. Intravenous anesthetics, particularly propofol, remain central due to their capacity to reduce cerebral metabolic rate, stabilize neuronal membranes and attenuate free radical formation. Propofol also exerts anti-inflammatory effects and limits lipid peroxidation, making it a valuable component of metabolic suppression during cooling, arrest and reperfusion [98].

Volatile anesthetics offer similar cytoprotective effects through preconditioning pathways, although their impact in vasoreactivity requires careful titration during selective perfusion.

Magnesium sulfate may mitigate excitotoxicity by antagonizing NMDA receptors and reducing calcium influx, while lidocaine stabilizes neuronal membranes and may reduce the burden of ischemia-induced arrhythmias and electrical instability. Dexmedetomidine provides neuroprotection through sympatholytic, anti-inflammatory and anti-apoptotic mechanisms and may facilitate smoother hemodynamic transitions during cooling and rewarming. Additional adjuncts, including corticosteroids, free-radical scavengers and agents targeting mitochondrial integrity, have been investigated, but their benefit remains context-dependent and less clearly defined [84,99,100].

### 3.4. Spinal Cord, Renal and Visceral Protection

**Figure 2:**
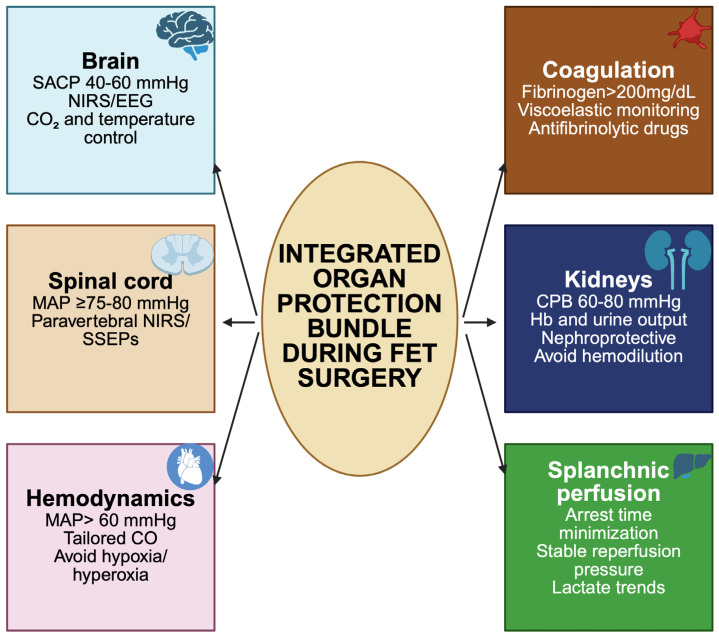
Integrated organ-protection bundle during FET surgery. SACP: selective antegrade cerebral perfusion; MAP: mean arterial pressure; NIRS: near-infrared spectroscopy; CO: cardiac output; CPB: cardiopulmonary bypass; Hb: hemoglobin; EEG: electroencephalogram; SSEP: somatosensory-evoked potentials. Created in BioRender. Pirri, C. (2026) https://BioRender.com/rnwb0xq (accessed on 17 December 2025).

Organ protection beyond the brain is a defining challenge of the FET procedure (Figure 2). The hybrid prosthesis extends into the proximal descending thoracic aorta, covering multiple segmental arteries and profoundly altering spinal cord, renal and splanchnic perfusion. During circulatory arrest and the subsequent ischemia–reperfusion sequence, these organs are exposed to abrupt changes in flow, temperature and metabolic demands. A comprehensive protection strategy must therefore anticipate regional vulnerabilities and implement tailored interventions to maintain adequate oxygen delivery throughout the operative course [101].

#### 3.4.1. Risk of Spinal Cord Ischemia in FET and Strategies to Maintain Spinal Cord Perfusion

Spinal cord ischemia represents a feared complication of FET surgery, driven by interruption of segmental arterial inflow, collapse of the collateral network and hypotension during or after circulatory arrest. Because spinal cord perfusion pressure depends on the gradient between mean arterial pressure (MAP) and intrathecal pressure, even modest reduction in MAP or increase in venous congestion can precipitate neurologic injury [14,84,102,103]. Strategies to protect the spinal cord include

- Maintaining adequate MAP targets, particularly during rewarming and early reperfusion, when autoregulation in unstable. Pressures ≥ 70–80 mmHg are commonly targeted once systemic flow is restored [104].
- Avoiding excessive hemodilution, as spinal cord oxygen extraction in highly sensitive to decrease in hemoglobin [14,105].
- Somatic or paravertebral NIRS monitoring, placed over the thoracic paraspinal regions, extends the utility of NIRS beyond the brain by providing a surrogate indicator of spinal cord perfusion. Declines in paravertebral rSO*_2_* can precede clinical or hemodynamic signs of spinal hypoperfusion and correlate with reduced flow within the intercostal and segmental arterial network, an important consideration during FET deployment, when the endovascular component may compromise spinal cord blood flow supply. These sensors assist in detecting early malperfusion, guiding interventions such as MAP augmentation, hemoglobin optimization, cerebrospinal fluid drainage or modification of distal perfusion strategies [14,84,106].
- Ensuring controlled rewarming, preventing vasodilation-induced hypotension that may reduce spinal cord perfusion pressure.
- Considering cerebrospinal fluid drainage in selected patients at high risk (e.g., extensive coverage of the descending aorta), although its use in FET remains institution-dependent [14,84,107].

Collectively, these strategies aim to maintain a stable spinal cord perfusion pressure during the vulnerable phases of arrest, graft deployment and reperfusion.

#### 3.4.2. Renal Protection Strategies

The kidneys are highly susceptible to ischemia–reperfusion injury during FET surgery due to interruption of distal perfusion, hemodilution, inflammatory activation and postoperative vasoplegia. Renal hypoperfusion is exacerbated by prolonged arrest times, low-flow states and venous congestion, making perioperative management a multidimensional challenge [108]. Key renal protection measures include

- Optimizing renal perfusion pressure, particularly during rewarming, where systemic vasodilation can reduce renal blood flow [109].
- Avoiding excessive hemodilution, maintaining hemoglobin levels adequate for oxygen delivery [110].
- Restricting crystalloid administration, limiting interstitial edema and renal congestion [111].
- Preventing hyperglicemia, acidosis and large temperature shifts, all of which can potentiate renal injury [112].
- Reducing nephrotoxic exposures, including careful avoidance of excessive vasoconstriction or nephrotoxic medications during vulnerable phases [113].

Renal protection requires a consistent balance between adequate perfusion pressure, controlled fluid management and timely detection of early functional compromise.

#### 3.4.3. Splanchnic and Visceral Perfusion During Circulatory Arrest

The splanchnic organs face a predictable ischemic period during circulatory arrest, followed by sudden reperfusion. This sequence promotes intestinal mucosal injury, endotoxin translocation, coagulopathy and systemic inflammatory response, all of which may worsen postoperative outcomes [114,115]. Given the absence of standardized protocols, protective interventions are generally guided by physiological principles and by indirect evidence derived from aortic and mesenteric-reperfusion models [116,117]. These measures include

- Ensuring adequate systemic perfusion before arrest, optimizing oxygen delivery and acid-base status.
- Minimizing the duration of circulatory arrest, especially in patients with impaired mesenteric vascular reserve.
- Maintaining stable perfusion pressures during early reperfusion, avoiding underfilling or vasodilatory hypotension.
- Controlling rewarming, since rapid thermal shifts can promote intestinal microcirculatory dysfunction.
- Preventing hyperoxia and excessive lactate accumulation, which may intensify oxidative injury during reperfusion.

Early postoperative vigilance remains essential, as visceral hypoperfusion often manifests through rising lactate, ileus or coagulopathy before progressing to overt organ failure.

### 3.5. Hemodynamic Management and Advanced Monitoring

**Figure 3:**
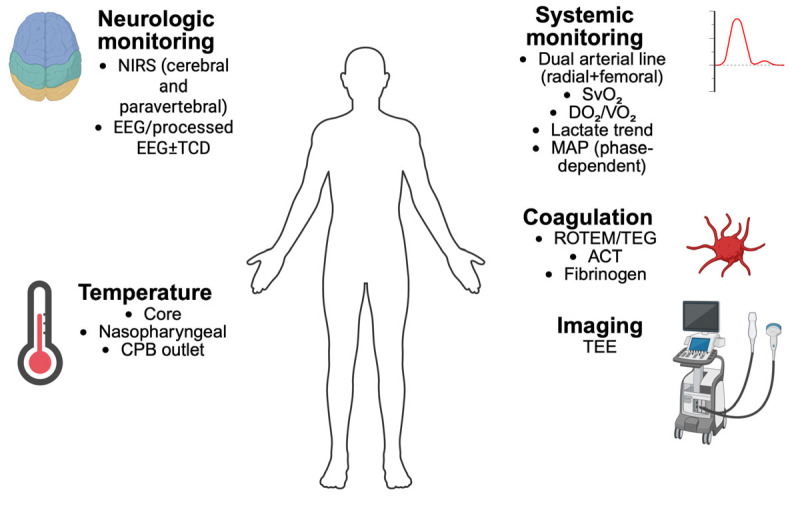
Comprehensive multimodal monitoring during FET surgery. NIRS: near-infrared spectroscopy; EEG: electroencephalogram; TCD: trans-cranial Doppler; CPB: cardiopulmonary bypass; SvO_2_: mixed venous oxygen saturation; DO_2_: oxygen delivery; VO_2_: oxygen consumption; MAP: mean arterial pressure; ROTEM: rotational thromboelastometry; TEG: thromboelastography; ACT: activated clotting time; TEE: tranesophageal echocardiography. Created in BioRender. Pirri, C. (2026) https://BioRender.com/k79hmp3 (accessed on 17 December 2025).

Hemodynamic management during FET surgery requires constant adaptation to rapid shifts in preload, afterload, vascular tone and cardiac function across the phase of cooling, circulatory arrest, reperfusion and rewarming. Because perfusion territories are intermittently redistributed between selective cerebral flow and global bypass support, the anesthesiologist must integrate multimodal data-arterial pressure monitoring, cardiac output indices, mixed venous saturation, lactate dynamics and transesophageal echocardiography (TEE) to maintain adequate systemic oxygen delivery and organ perfusion. Precision in hemodynamic control is central to reducing ischemic complications, ensuring spinal cord and renal protection and supporting cerebral perfusion during SACP (Figure 3).

#### 3.5.1. Invasive Monitoring and Arterial Pressure Interpretation

Dual-site arterial pressure monitoring is fundamental in FET surgery.

Radial arterial pressure may significantly diverge from central aortic pressure during CPB, deep hypothermic circulatory arrest and retrograde perfusion, whereas femoral pressure often better approximates central pressure in these phases [118,119,120].

In aortic arch surgery with axillary or innominate cannulation, right radial pressure is additionally influenced by selective antegrade flow and may overestimate true proximal aortic pressure [121]. These data support the use of combined upper and lower body arterial monitoring to detect perfusion gradients and balance cerebral and systemic perfusion throughout the procedure [122].

Additional monitoring such as central venous pressure, pulmonary artery catheterization and NIRS helps refine the assessment of venous congestion, right ventricular load and regional oxygen delivery during complex cardiac procedures, including aortic arch surgery [123,124].

#### 3.5.2. Perfusion Metrics: SvO_2_, Lactate, DO_2_/VO_2_

Standard measures such as mean arterial pressure are insufficient alone to estimate systemic perfusion during circulatory arrest or selective cerebral perfusion. Advanced monitoring provides a more comprehensive perspective.

- Mixed venous oxygen saturation (SvO*_2_*) and central venous oxygen saturation (ScvO*_2_*) reflect global balance between oxygen delivery and consumption, particularly during reduced flow states and early reperfusion [125].
- Lactate trends provide a sensitive index of systemic hypoperfusion and ongoing anaerobic metabolism, especially relevant after periods of distal ischemia during arch reconstruction [126,127].
- Oxygen delivery and consumption (DO*_2_*/VO*_2_*): the DO*_2_*/VO*_2_* relationship offers a sensitive metric of systemic perfusion adequacy. When DO*_2_* falls below the critical threshold, common during low-flow SACP, circulatory arrest transitions or early reperfusion, VO*_2_* becomes supply-dependent, leading to increased extraction and falling SvO*_2_*/ScvO*_2_*. Rising VO*_2_*/DO*_2_* ratios help identify occult hypoperfusion despite apparently adequate MAP, prompting adjustments in pump flow, hemoglobin or vasopressor therapy [125].

Dynamic interpretation of these metrics support titration of vasopressors, inotropes, perfusion flows and fluid therapy according to evolving metabolic demand.

#### 3.5.3. Role of Transesophageal Echocardiography (TEE)

TEE is indispensable throughout all phases of FET surgery. Preoperatively, it defines ventricular function, aortic pathology, true lumen versus false lumen perfusion in dissection and the presence of thrombus or aortic insufficiency. During the procedure, TEE ensures positioning of the guidewire and stented graft, verifies laminar antegrade flow after deployment and identifies complications such as dynamic obstruction, malposition or new entry tears, although visualization of the distal descending aorta and complete stent graft expansion may still require adjunctive fluoroscopy or angiography.

TEE also guides hemodynamic management by providing real-time assessment of preload adequacy, right ventricular function during reperfusion, left ventricular recovery during rewarming, air embolism following deairing and graft manipulation [128].

Its integration with perfusion parameters and arterial pressure monitoring enables an individualized, physiology-based approach.

### 3.6. Anesthetic Techniques and Pharmacologic Management

Anesthetic management in FET surgery aims to maintain cerebral and systemic perfusion stability, provide metabolic suppression during periods of hypothermia and arrest and attenuate the inflammatory and hemodynamic consequences of reperfusion. The choice of anesthetic technique integrates protection, cardiovascular stability and organ-specific vulnerabilities [129].

#### 3.6.1. TIVA vs. Volatile-Based Strategies

Both total intravenous anesthesia (TIVA) and volatile anesthetics are widely used in aortic arch and FET surgery. Although no randomized or comparative studies have specifically evaluated these techniques in the setting of arch or FET procedures, the safety and efficacy of both regimens are indirectly supported by randomized trials and meta-analyses conducted in other forms of major cardiac surgery, which have not demonstrated consistent superiority of one approach over the other [130,131]. As a result, anesthetic choice in arch surgery relies primarily on physiological rationale, neuromonitoring requirements and institutional practice.

TIVA, typically propofol-based, provides predictable metabolic suppression and decreases cerebral metabolic rate. Propofol also exerts antioxidant and anti-inflammatory effects relevant during cooling, circulatory arrest and reperfusion and its pharmacokinetic stability under hypothermia facilitates titration across transitions in perfusion flow and temperature [98]. Moreover, TIVA facilitates EEG-guided titration during the cooling phase, allowing predictable progression toward burst suppression or electrocerebral silence before circulatory arrest, when drug delivery to the brain is still reliable. During arrest, metabolic suppression is maintained by the combined effect of hypothermia and pre-induced EEG depression [132].

Volatile anesthetics provide established ischemic-preconditioning effects through mitochondrial KATP channels activation and modulation of excitotoxic and inflammatory pathways, potentially enhancing tissue tolerance to reperfusion injury [133]. However, their cerebral vasodilatory properties may alter cerebrovascular resistance and complicate autoregulation during selective antegrade cerebral perfusion, requiring cautious titration to avoid flow maldistribution [134]. Given the absence of arch-specific comparative data, both TIVA and volatile-based techniques can be used safely when integrated within a multimodal protective strategy.

#### 3.6.2. Perioperative Management of Vasoplegia

Vasoplegia is common after cardiopulmonary bypass and particularly pronounced in FET surgery due to prolonged hypothermia, ischemia–reperfusion injury, hemodilution and systemic inflammation. The condition is characterized by reduced systemic vascular tone, hypotension refractory to catecholamines and adequate or elevated cardiac output. Management combines optimization of volume status, correction of metabolic and inflammatory triggers and targeted vasoactive therapy [135].

Norepinephrine remains the first-line agent for restoring vascular tone and maintaining MAP during selective cerebral and systemic reperfusion. Vasopressin is highly effective. In vasoplegia related to CPB or inflammatory vasodilation, restoring responsiveness in catecholamine-resistant hypotension without significantly increasing pulmonary vascular resistance. Methylene blue may be used in refractory cases by inhibiting the nitric oxide c-GMP pathway, though caution is required in patients with serotonergic therapies. Hydroxocobalamin represents an alternative rescue agent, scavenging nitric oxide and hydrogen sulfide, with growing clinical adoption [136]. Angiotensin II may be considered in severe vasoplegia unresponsive to conventional therapy, restoring vasomotor tone through renin-angiotensin pathway activation [137].

Early identification and aggressive management of vasoplegia are essential to maintain cerebral perfusion pressure, spinal cord perfusion and renal oxygen delivery during the hemodynamic transitions that define FET surgery.

### 3.7. Coagulation and Hemostasis Management

Coagulation management represents a critical component of perioperative care in FET surgery, as profound hypothermia, extended CPB, hemodilution and extensive surgical surfaces converge to produce complex coagulopathies. Early identification of coagulation deficits and timely correction are central to limiting transfusion requirements, reducing postoperative bleeding and improving overall outcomes [138].

#### 3.7.1. Mechanisms of Coagulopathy During FET Surgery

FET surgery induces a multifactorial coagulopathy driven by the combined effects of hypothermia, hemodilution, CPB-induced inflammatory activation and consumption of coagulation factors. Hypothermia impairs platelet function, reduces enzymatic activity within the coagulation cascade and alters fibrin polymerization. Hemodilution during CBP lowers circulating levels of fibrinogen, platelets and coagulation factors, while the cardiopulmonary circuit promotes contact activation and fibrinolysis. Prolonged surgical exposure of mediastinal and aortic surfaces further contributes to blood loss and factor consumption. Reperfusion and rewarming introduce additional challenges, including transient platelet dysfunction and vasodilatory states that exacerbate ongoing bleeding [138,139]. Understanding these dynamic changes is essential for guiding targeted hemostatic therapy.

#### 3.7.2. Role of Viscoelastic Testing (ROTEM/TEG)

**Figure 4:**
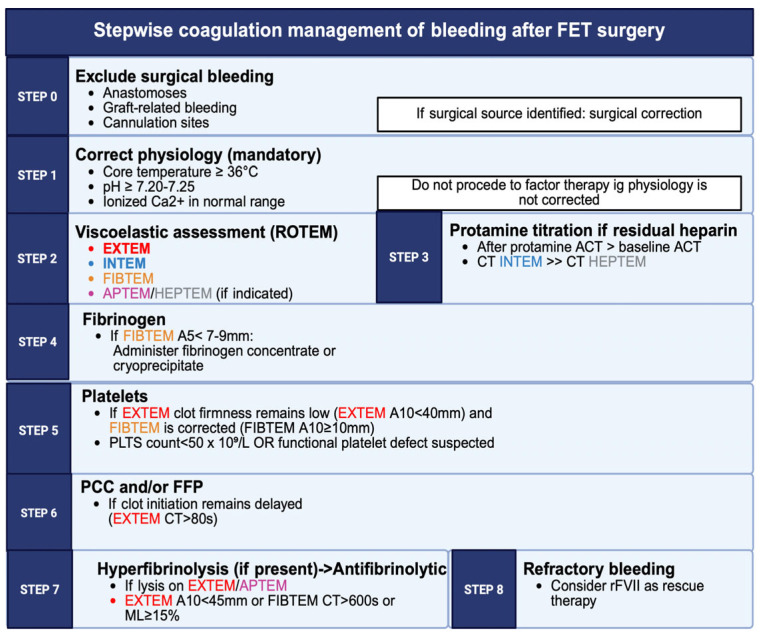
Stepwise, goal-directed management of postoperative bleeding after frozen elephant trunk surgery. FET: frozen elephant trunk; ACT: activated clotting time; EXTEM: extrinsic pathway thromboelastometry; INTEM: intrinsic pathway thromboelastometry; APTEM: aprotinin-modified thromboelastometry; HEPTEM: heparinase-modified thromboelastometry; PCC: prothrombin complex; FFP: fresh frozen plasma; rFVII: recombinant activated factor VII; A5: amplitude at 5 min; A10: amplitude at 10 min; CT: clotting time; ML: maximum lysis; PLTS: platelets. Created in BioRender. Pirri, C. (2026) https://BioRender.com/fl69gv3 (accessed on 17 December 2025).

Viscoelastic testing has become central to modern coagulation management during complex aortic surgery. ROTEM and TEG provide real-time assessment of clot formation, strength, fibrinolysis and platelet function, offering a more comprehensive overview than conventional laboratory tests. These assays allow rapid identification of specific deficits, such as hypofibrinogemia, platelet dysfunction or hyperfibrinolysis, enabling tailored interventions with fibrinogen concentrate, cryoprecipitate, platelets or antifibrinolytics. In addition to platelet dysfunction and fibrinolysis, FET surgery is characterized by progressive loss and dilution of soluble coagulation factors, particularly factors V, VII and von Willebrand factor, due to hypothermia, hemodilution and CPB circuit consumption [138]. Viscoelastic assays help detect these deficits early, allowing targeted replacement through plasma components or factor concentrates when indicated. The use of these tests has been associated with reduced transfusion requirements, improved correction of coagulopathy and decreased re-exploration for bleeding [15,140]. In FET procedures, where hypothermia and extended CPB times significantly distort standard laboratory values, viscoelastic testing is particularly valuable for guiding goal-directed therapy (Figure 4).

#### 3.7.3. Fibrinogen Management and Platelet Optimization

Fibrinogen depletion is a hallmark of coagulopathy following CPB and hypothermic arrest. Early replacement is essential, as fibrinogen is the first coagulation factor to fall below critical thresholds and plays a central role in clot firmness and stability. Current practice favors goal-directed replacement using fibrinogen concentrate or cryoprecipitate when functional assays such as FIBTEM indicate low clot amplitude. Targeting fibrinogen levels of 200–250 mg/dL or FIBTEM values within institutional ranges helps restore adequate hemostasis [141,142,143]. Platelet dysfunction is equally prominent due to hypothermia, CPB-induced activation and exhaustion and hemodilution. Platelet transfusion should be guided by quantitative and functional data, including viscoelastic markers of clot strength (e.g., MCF/MA) [144]. Maintenance of normothermia during rewarming and avoidance of excessive crystalloid infusion support platelet function and minimize dilutional thrombocytopenia.

Beyond fibrinogen depletion, significant loss and consumption of coagulation factors occur during CPB and hypothermic circulatory arrest, particularly factors V, VIII and components of von Willebrand complex. Hemodilution, circuit adsorption and hypothermia-induced impairment of enzymatic activity further compromise thrombin generation. Replacement of soluble clotting factors via plasma, prothrombin complex concentrates or factor concentrates where appropriate should therefore be guided by viscoelastic signatures suggestive of impaired coagulation cascade activity rather than isolated laboratory thresholds. This approach ensures restoration of both fibrin polymerization and thrombin-mediated clot propagation [145,146].

#### 3.7.4. Antifibrinolytic Therapy

Antifibrinolytic therapy represents a key component of bleeding prevention in FET surgery. Although dedicated studies in aortic arch or FET procedures are limited, evidence from thoracic aortic surgery and broader cardiac surgery consistently shows that tranexamic acid reduces plasmin-mediated fibrinolysis, stabilizes clot formation under hypothermic and inflammatory conditions and decreases perioperative blood loss and transfusion requirements [147,148]. Tranexamic acid administration during induction and throughout CPB is therefore widely adopted as part of multimodal blood conservation strategies in complex aortic repair [147,148]. Aprotinin is a potent antifibrinolytic agent with demonstrated efficacy in reducing bleeding during high-risk cardiac and aortic surgery; however, its use has declined following concerns raised in large clinical trials [149]. Lysin analogs such as tranexamic acid remain the preferred option in most contemporary centers. Because FET surgery is frequently associated with CPB-induced activation of fibrinolysis-exacerbated by hypothermia, extensive surgical surfaces and prolonged perfusion, continuous surveillance of fibrinolytic activity is essential. Viscoelastic assays allow early identification of hyperfibrinolysis, with parameters such as LY30 or maximum lysis (ML) providing actionable thresholds for timely tranexamic acid supplementation or escalation of therapy [150,151]. This goal-directed approach helps prevent refractory coagulopathic bleeding in the high-risk perioperative environment typical of FET reconstruction.

#### 3.7.5. Blood Sparing Strategies

Blood conservation is essential in FET surgery to limit transfusion-related complications and preserve perioperative hemostasis. Retrograde autologous priming (RAP) and minimized CPB circuits reduce hemodilution and help maintain higher hematocrit during complex aortic procedures [152,153]. Intra-operative cell salvage is particularly valuable in extensive mediastinal and aortic dissections, decreasing allogenic transfusion requirements in thoracic aortic surgery [154,155]. Ultrafiltration techniques, including modified ultrafiltration, improve hemoconcentration, remove inflammatory mediators and support hemostatic recovery before separation from CPB [156]. Restrictive crystalloid administration prevents dilutional coagulopathy and reduces interstitial edema, a key determinant of bleeding in aortic surgery [157]. Finally, meticulous surgical hemostasis, including the use of topical agents and precise control of arterial inflow during graft manipulation, remains fundamental in optimizing blood conservation during arch and FET reconstruction [139].

### 3.8. Postoperative Management

Post-operative care following FET surgery requires structured, multidisciplinary surveillance to detect early complications related to neurologic injury, organ malperfusion, reperfusion syndromes, hemodynamic instability and residual coagulopathy. Management focuses on preventing secondary insults, supporting end-organ recovery and facilitating early mobilization within a monitored environment.

#### 3.8.1. Neurologic Surveillance

Neurologic assessment is critical in the early postoperative period, when impairments may reflect embolic events, hypoperfusion during arrest, hyperperfusion after rewarming or spinal cord ischemia. Serial examinations, pupillary responses, motor strength and level of consciousness are performed as sedation is weaned. Continuous NIRS monitoring may provide adjunctive information on cerebral oxygenation during hemodynamic transitions. Early detection of spinal cord ischemia relies on frequent lower limb motor testing, monitoring paravertebral NIRS trends when available and maintaining adequate perfusion pressure. Suspicious findings warrant prompt escalation, including MAP augmentation or cerebrospinal fluid drainage [74,84,158,159].

#### 3.8.2. Renal and Hemodynamic Monitoring

Renal function is particularly vulnerable after distal ischemia and reperfusion. Postoperative management emphasizes maintaining adequate perfusion pressure, avoiding venous congestion and monitoring urine output, creatinine trends and lactate clearance. Doppler-based indices, together with early AKI biomarkers, may assist in identifying early renal perfusion impairment [110,160].

Hemodynamic goals focus on securing stable MAP without excessive afterload, supporting right ventricular function as venous return increase and preventing hypotension that could jeopardize cerebral or spinal cord perfusion. Vasopressor requirements are reassessed continuously, as vasoplegia may persist or emerge after rewarming and anesthesia washout.

#### 3.8.3. Ventilation and Oxygenation Strategies

Ventilatory management aims to minimize pulmonary stress while supporting adequate oxygen delivery. Protective ventilation with low tidal volumes, moderate PEEP and avoidance of hyperoxia helps limit oxidative stress injury during reperfusion. Gradual normalization of CO*_2_* is essential after hypothermic phases to prevent abrupt shifts in cerebral blood flow. Timely extubation is pursued once hemodynamic, neurologic and respiratory criteria are met, as prolonged mechanical ventilation increases the risk of delirium, infection and impaired mobilization [161,162].

#### 3.8.4. Coagulation Reassessment and Bleeding Control

Postoperative bleeding may reflect residual hypothermia-related dysfunction, incomplete factor replacement or surgical sources. Early reevaluation with ROTEM modules (EXTEM, FIBTEM and HEPTEM) provides rapid insight into fibrinogen status, platelet contribution to clot firmness and potential residual heparin effect. Targeted correction with fibrinogen concentrates, platelets or plasma components is instituted as indicated. Persistent or unexpected bleeding requires prompt imaging or surgical evaluation to exclude graft-related or anastomotic complications. Avoiding crystalloid overload is important to prevent dilutional coagulopathy and maintain effective clot formation [140].

#### 3.8.5. Pain Management and Early Mobilization

Effective analgesia supports respiratory mechanics, reduces sympathetic activation and facilitates early mobilization. Multimodal regimens, opioids, acetaminophen and non-opioid adjunct when appropriate are preferred to minimize sedation and hemodynamic fluctuations. Early mobilization, tailored to hemodynamic stability and neurologic status, reduces risk of pulmonary complications, venous thromboembolism and deconditioning. Mobilization also aids neurologic assessment, particularly regarding lower-limb strength and gait, which may reveal subtle deficits suggestive of spinal cord hypoperfusion [163,164].

### 3.9. Outcomes

Outcomes after FET surgery reflect the interplay between surgical technique, perfusion strategy, patient comorbidities and perioperative management. Despite advances in cerebral protection, temperature modulation and end-organ surveillance, neurologic injury, distal organ dysfunction and early mortality remain significant considerations. The following sections summarize contemporary evidence regarding postoperative neurologic outcomes, mortality and major morbidities and predictors of adverse events.

#### 3.9.1. Neurologic Outcomes

Neurologic complications remain of the primary determinants of postoperative recovery following FET surgery. Stroke rates vary across series but generally range from 3% to 10%, influenced by embolic burden, adequacy of selective cerebral perfusion and complexity arch reconstruction. Temporary neurologic dysfunction, including delirium, cognitive slowing or agitation, appears more frequently and is often related to multifactorial contributors such as anesthesia washout, inflammation and cerebral autoregulation disturbances during rewarming [165,166].

Spinal cord injury, although less common, represents one of the most devasting complications. Reported incidence ranges from 2% to 8%, with higher risk in cases involving extensive stent-graft coverage of the descending aorta, prolonged circulatory arrest or insufficient postoperative perfusion pressure. Early recognition and aggressive management, particularly maintaining spinal cord perfusion pressure, are essential to improving neurologic recovery [14,167].

#### 3.9.2. Mortality and Major Morbidities

Early mortality after FET surgery typically ranges from 5% to 15%, depending on the indication (acute dissection vs. chronic pathology), urgency of the procedure and preoperative hemodynamic status. Evidence consistently demonstrates higher mortality in patients undergoing emergent surgery for acute type A dissection or in those presenting with malperfusion syndrome [168].

Major postoperative morbidities include

- Acute kidney injury, which develops in up to one-third of patients and is strongly associated with distal ischemia time, reperfusion injury and systemic inflammation [169].
- Respiratory complications, including prolonged ventilation and pneumonia, particularly in older or frail patients [170].
- Coagulopathy and significant bleeding, often requiring transfusion or re-exploration [171].

## 4. Discussion

The management of patients undergoing FET surgery continues to evolve as surgical techniques, perfusion strategies and monitoring technologies advance. This review highlights how contemporary anesthetic practice has shifted from protocol-driven, temperature-dominated approaches toward individualized, physiology-based management that integrates cerebral, spinal, renal and systemic protection [3,9,10,17,18]. The complexity of these procedures stems from the unique circulatory environments created by selective antegrade cerebral perfusion, hypothermic arrest and staged reperfusion conditions that challenge conventional intraoperative decision-making and demand continuous adaptation [19,35,36,39,40,45,46].

One of the central themes emerging from recent evidence is the importance of dynamic perfusion management. Rather than relying on fixed flow or pressure targets, modern strategies emphasize real-time interpretation of multimodal data, cerebral oximetry, EEG patterns, Doppler findings, arterial pressure gradients and metabolic indices. This shift mirrors broader trends in cardiac anesthesia, where individualized perfusion has replaced one-size-fits-all techniques [74,82,83,84,85,86,87,88,89]. In FET surgery, where even brief deviations in cerebral or distal organ perfusion may have lasting consequences, such an approach is particularly relevant. The benefit is evident in declining neurologic injury rates at high-volume centers that have adopted integrated monitoring algorithms [7,10,75,76,77,78,79,80]. There is another consistent observation in the growing recognition of organ interdependence. Improvements in cerebral protection, for example, must not occur at the expense of spinal cord or renal perfusion, particularly as FET devices extend further into the descending aorta [11,12,13,14,101,102,103,104,105,106,107]. This interdependence underscores the need for continuous hemodynamic stability during transitions between selective and systemic perfusion, not merely to protect the brain but to preserve the broader metabolic landscape required for postoperative recovery. Spinal cord ischemia, although infrequent, remains a devasting complication whose prevention hinges on meticulous control of perfusion pressure, hemoglobin concentration and rewarming dynamics, in addition to surgical consideration such as graft length and distal coverage [14,84,102,103,104,105,106,107].

Coagulation management represents another domain in which anesthetic practice has undergone substantive refinement. Hypothermia-induced coagulopathy and factor depletion are intrinsic to the procedure, yet the expansion of viscoelastic testing has enabled earlier and more precise correction of fibrinogen deficiency, platelet dysfunction and hyperfibrinolysis [15,16,138,139,140,141,142,143,144,145,146]. This evolution aligns with a broader movement toward blood-sparing strategies that minimize transfusion-related morbidity. While standardized algorithms vary among institutions, the consistent message across the literature is that goal-directed hemostatic therapy improves both bleeding control and postoperative outcomes [152,153,154,155,156,157].

Despite these advances, several challenges persist. Heterogeneity in cannulation strategies, variability in perfusion targets and differences in neuromonitoring availability contribute to wide inter-institutional variation in practice [19,20,21,22,23,24,25,26,27,28,29,30,31,32]. Moreover, data on long-term neurologic and organ-specific outcomes remain limited, as most published series focus on in-hospital or early postoperative endpoints [165,166,167,168,169]. The increasing use of FET in acute type A dissection introduces further complexity, as these patients often arrive with pre-existing malperfusion or hemodynamic instability that complicates intraoperative management and blunts the benefits of optimal perfusion and monitoring strategies [11,12,13].

Emerging technologies, such as advanced microcirculatory monitoring, machine learning-based perfusion prediction and next-generation hybrid grafts with optimized flow characteristics may help address some of these gaps. However, their integration into practice will require standardized definitions, multicenter collaboration and a refined understanding of which metrics truly correlate with clinically relevant outcomes [172,173,174,175,176].

Additionally, the expanding adoption of moderate hypothermia paired with robust selective cerebral perfusion indicates a paradigm shift that reduces the metabolic penalties of deep hypothermia, but further data are needed to delineate which patient subgroups may still benefit from more profound cooling [9,10,55,56,57,58,59,60,61,62,63,64].

Finally, it is increasingly clear that optimal outcomes depend not on individual interventions but on the coherence of the entire perioperative strategy. High-performing centers consistently demonstrate structured pathways for preoperative assessment, intraoperative perfusion management, neuromonitoring integration and postoperative surveillance, suggesting that system-level organization may influence outcomes as strongly as any single technique [17,18,74,82,138,158,159,160,161,162,163,164]. Multidisciplinary coordination, between anesthesiologist surgeons, perfusionists and critical care teams, remains the cornerstone of safe and effective FET management.

## 5. Conclusions

The perioperative management of patients undergoing FET surgery has evolved toward an individualized, physiology-based approach that integrates multimodal neuromonitoring, organ-specific perfusion strategies and goal-directed hemostasis. Advances in selective cerebral perfusion, temperature modulation and viscoelastic-guided coagulation therapy have contributed to reducing neurologic injury and major postoperative morbidity. However, significant variability in practice persists among institutions, reflecting heterogenous perfusion strategies, monitoring availability and patient profiles, particularly in acute aortic dissection. Overall, optimal outcomes rely not on isolated interventions but on coherent, team-based pathways that maintain cerebral, spinal and systemic perfusion throughout all phases of repair.

## 6. Future Directions

Future progress in the anesthetic management of FET surgery will depend on more precise characterization of end-organ vulnerability and improved tools for real-time physiologic assessment. Advances in microcirculatory monitoring, including tissue oximetry beyond traditional NIRS and emerging perfusion imaging technologies, may allow earlier detection of occult hypoperfusion during selective cerebral perfusion and reperfusion. Artificial intelligence-driven decision support system, incorporating hemodynamic, neurologic and metabolic fata, hold promise for predicting perfusion thresholds and guiding individualized flow and pressure targets. Further refinement of temperature and perfusion strategies will require multicenter studies capable of defining optimal hypothermic ranges for different patient subsets and clarifying the role of metabolic suppression in moderate hypothermia. Parallel efforts are needed to standardize viscoelastic-guided hemostatic algorithms and to evaluate factor-based approaches that minimize transfusion exposure. While viscoelastic-guided transfusion algorithms based on ROTEM are increasingly adopted in cardiovascular surgery, their specific validation in FET procedures remains limited. Given the extreme physiologic alterations (deep or moderate hypothermia, extended ischemia–reperfusion), tailored ROTEM thresholds and factor-based protocols for this subgroup are still under development.

Finally, the integration of next-generation hybrid grafts designed to preserve spinal cord blood supply may shift current paradigms in distal perfusion protection.

A coordinated research agenda, combining surgical innovation, perfusion science and advanced neuromonitoring, will be essential to enhance neurologic and systemic outcomes in this complex and evolving field.

## Figures and Tables

**Table 1:** Cannulation strategies in frozen elephant trunk surgery: perfusion, characteristics, advantage and limitations. SACP: selective antegrade perfusion; CPB: cardiopulmonary bypass; FET: frozen elephant trunk.

Cannulation Site	Flow Direction	Cerebral Perfusion Characteristics	Lower Body Perfusion	Main Advantages	Risks	Typical Indications
Right axillary artery [20,21,22,23]	Antegrade	Reliable antegrade cerebral perfusion; supports unilateral SACP; depends on circle of Willis competence	Limited during circulatory arrest	Reduced embolic risk; physiological flow direction; facilitates SACP	Risk of local vascular injury; potential flow limitation at high CPB flows	Standard approach in elective and emergency arch/FET surgery
Innominate artery [19]	Antegrade	Direct bilateral cerebral perfusion when SACP is established primarily	Limited during circulatory arrest	Central, high-flow access; effective cerebral protection	Surgical exposure required; not feasible in all anatomies	Total arch replacement with proximal vessel control
Left axillary artery [24,25]	Antegrade	Preferential left hemispheric perfusion; dependent on collateral circulation	Limited during circulatory arrest	Alternative when right-sided access is not feasible	Risk of left arm ischemia; reliance on circle of Willis	Right-sided vascular disease or dissection
Bilateral axillary arteries [26]	Antegrade	Symmetric bilateral cerebral perfusion; minimizes hemispheric flow asymmetry	Limited unless combined with distal cannulation	Enhanced cerebral homogeneity during prolonged SACP	Increased technical complexity; higher risk of local complications	High-risk patients; prolonged circulatory arrest; incomplete Willis
Femoral artery [27,28]	Retrograde	Indirect cerebral perfusion; higher embolic potential	Effective lower body perfusion	Rapid access, high-flow capability	Retrograde embolization; false lumen perfusion in dissection	Rescue or adjunctive cannulation; pre-existing malperfusion
Central aortic cannulation [29,30]	Antegrade	Physiological antegrade flow	Effective lower body perfusion	Direct central access; good flow dynamics	Limited feasibility in arch pathology; surgical constraints	Selected elective cases
Dual arterial cannulation [31]	Antegrade + retrograde	Stable cerebral perfusion via axillary inflow	Preserved distal organ perfusion	Balanced cerebral and systemic perfusion	Increased stroke and renal risk; complex management	Preoperative malperfusion; prolonged arrest

## Data Availability

The original contributions presented in this study are included in the article. Further inquiries can be directed to the corresponding author.

## Abbreviations

The following abbreviations are used in this manuscript:

FET	Frozen elephant trunk
NIRS	Near-infrared spectroscopy
SACP	Selective antegrade cerebral perfusion
AKI	Acute kidney injury
SCI	Spinal cord injury
CPB	Cardiopulmonary bypass
